# Supplementary figures and images for: Resting-State Functional MRI Adaptation with Attention Graph Convolution Network for Brain Disorder Identification
Source: Brain Sci. 2022 Oct 20;12(10):1413. doi: 10.3390/brainsci12101413 (PMC9599902; doi:10.3390/brainsci12101413)

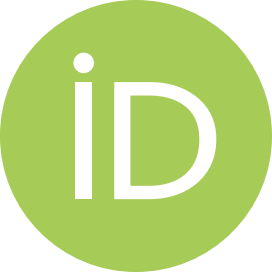

Supplement: Supplementary file 1 [file brainsci-12-01413-s001.zip › Definitions/logo-orcid.pdf]

● ACC(%)    ■ AUC(%)

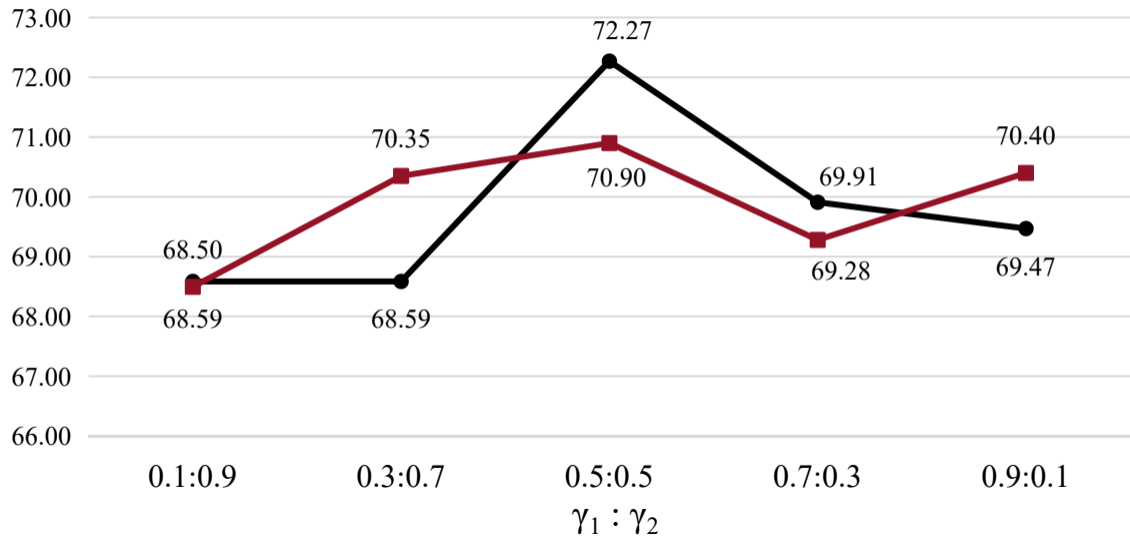

Supplement: Supplementary file 1 [file brainsci-12-01413-s001.zip › figs/figS1.pdf]

■ ACC(%)      ■ AUC(%)

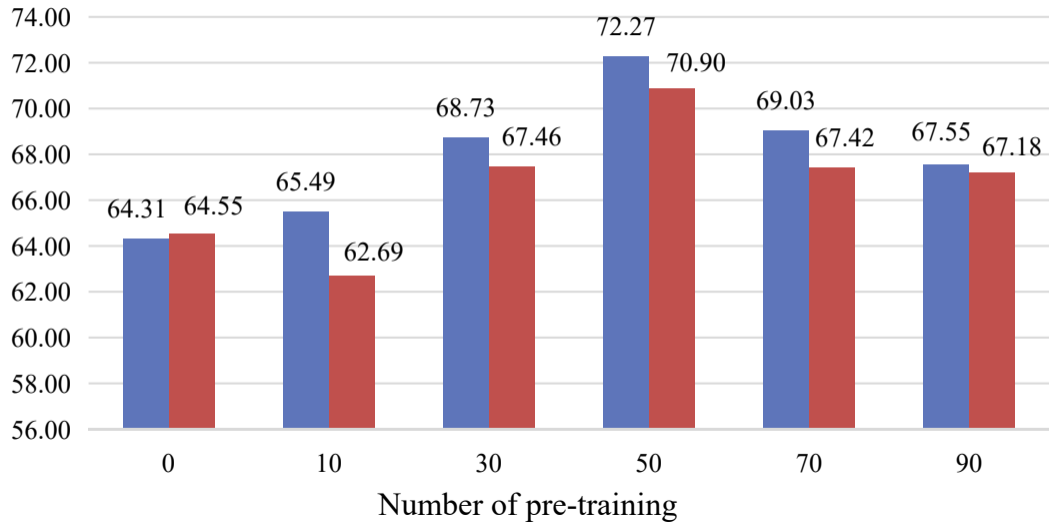

Supplement: Supplementary file 1 [file brainsci-12-01413-s001.zip › figs/figS2.pdf]
